# A New Optimized Version of a Colorectal Cancer-Targeted Immunotoxin Based on a Non-Immunogenic Variant of the Ribotoxin α-Sarcin

**DOI:** 10.3390/cancers15041114

**Published:** 2023-02-09

**Authors:** Javier Narbona, Rubén G. Gordo, Jaime Tomé-Amat, Javier Lacadena

**Affiliations:** 1 Department of Biochemistry and Molecular Biology, Faculty of Chemical Sciences, Complutense University, 28040 Madrid, Spain; 2 Centre for Plant Biotechnology and Genomics (UPM-INIA), Universidad Politécnica de Madrid, Pozuelo de Alarcón, 28223 Madrid, Spain

**Keywords:** immunotoxin, ribotoxin, non-immunogenic α-sarcin, furin, in vivo antitumor efficacy, colorectal cancer

## Abstract

**Simple Summary:**

Antitumor therapy with immunotoxins is limited by problems of immunogenicity and low efficacy in solid tumors. The different strategies to solve these problems include obtaining non-immunogenic variants of the toxins used as well as optimizing their release into the cytosol to increase their cytotoxic efficacy. Immunotoxins based on fungal ribotoxins have shown high specificity and antitumor efficacy. The aim of this work was to obtain two immunotoxins based on a non-immunogenic variant of the sarcin ribotoxin, one of which included a furin cleavage site. The results confirmed the null activation of the immunogenic response as well as a high antitumor efficacy of the optimized variant.

**Abstract:**

Due to its incidence and mortality, cancer remains one of the main risks to human health and lifespans. In order to overcome this worldwide disease, immunotherapy and the therapeutic use of immunotoxins have arisen as promising approaches. However, the immunogenicity of foreign proteins limits the dose of immunotoxins administered, thereby leading to a decrease in its therapeutic benefit. In this study, we designed two different variants of non-immunogenic immunotoxins (IMTXA33αSDI and IMTXA33furαSDI) based on a deimmunized variant of the ribotoxin α-sarcin. The inclusion of a furin cleavage site in IMTXA33furαSDI would allow a more efficient release of the toxic domain to the cytosol. Both immunotoxins were produced and purified in the yeast *Pichia pastoris* and later functionally characterized (both in vitro and in vivo), and immunogenicity assays were carried out. The results showed that both immunotoxins were functionally active and less immunogenic than the wild-type immunotoxin. In addition, IMTXA33furαSDI showed a more efficient antitumor effect (both in vitro and in vivo) due to the inclusion of the furin linker. These results constituted a step forward in the optimization of immunotoxins with low immunogenicity and enhanced antitumor activity, which can lead to potential better outcomes in cancer treatment.

## 1. Introduction

Among different types of cancer, colorectal cancer remains third in incidence and second in mortality when considering both sexes [1]. Colorectal cancer can be caused by genetic factors, which represent 5% of the total cases, while the remaining 95% are caused by sporadic or environmental factors [2]. Medical treatment for cancer usually consists of surgery with adjuvant chemotherapy or radiotherapy [3]. However, these adjuvant treatments are non-specific and lead to systemic toxicity, thereby affecting the overall health of patients. The greater usage of immunotherapy and monoclonal antibodies has led to more specific therapeutics and the design of more personalized treatments based on a patient’s own immune system and the antigenic profile of tumors, which express different tumor-associated antigens (TAAs) [4,5].

Immunotoxins are chimeric molecules that consist of a target domain (usually an antibody or a smaller fragment derived from it) that directs the action of a toxic domain (usually a protein obtained from plants or bacteria) [6]. Initially, immunotoxins consisted of whole antibodies fused with complete toxins, which led to inconveniences derived from the heterogeneity in their synthesis and off-target toxicities [7,8]. With the advent of DNA recombinant technology and the evolution of and improvements in their designs, recombinant single-chain immunotoxins containing both the marker and the toxic domain were developed that showed benefits of homogeneity in the production and better penetration of solid tumors due to their smaller size [9].

However, when considering the safety and potential therapeutic use of immunotoxins, some issues must be addressed. First, the selection of the TAA should be an antigen that is restricted to the tumoral tissue or is mostly absent in healthy tissues to avoid unspecific toxicities due to the binding of the immunotoxin to normal cells [10,11]. In this sense, the glycoprotein A33 (GPA33) is an integral membrane protein that is overexpressed in 95% of primary and metastatic colorectal cancer and is almost absent in healthy tissues [12,13]. This antigen, which is stably expressed in the cell membrane, shows a high degree of internalization after the binding of antibodies and the consequent formation of the antigen–antibody complex [14]. Thereby, GPA33 constitutes an ideal antigen for the development of colorectal cancer-directed immunotoxins and thus was employed as the target of different immunotoxins already designed by our group [15,16,17,18,19,20].

The immunogenic nature of bacterial and plant toxins represents a major drawback to their clinical use. The non-human nature of the immunotoxin is recognized as foreign by the patient’s immune system, thereby leading to the formation of antidrug antibodies (ADAs) that neutralize the immunotoxin administered, accelerate its clearance from the blood, and prevent further treatment [6,21]. Moreover, ADAs can cause immune-related adverse events such as infusion-related reactions, allergic reactions, anaphylaxis, delayed hypersensitivity, and autoimmunity [22,23]. Due to these immune adversities, toxic moieties from different sources such as fungal or human RNases are now being researched [24,25].

Ribotoxins stand out within the family of extracellular RNases due to their high thermostability, resistance to proteases, small size, poor immunogenicity, and highly effective enzymatic activity [8,26,27]. Among the ribotoxin family, α-sarcin is the most characterized member of this family. It exhibits an exquisite specific ribonucleolytic activity against one single rRNA phosphodiester bond located at the sarcin/ricin loop (SRL) in the larger subunit of the ribosomal RNA that leads to protein biosynthesis inhibition and death cell via apoptosis [24,28].

With the idea of designing new optimized variants of immunotoxins that lack immunogenic epitopes and the subsequent possibility of administrating several therapeutic doses without triggering the patient´s immune response to improve their clinical use, we designed and characterized (both in vitro and in vivo) two new non-immunogenic immunotoxins. The first was IMTXA33αSDI, which contained as the toxic domain a deimmunized form of α-sarcin lacking CD4^+^ T-cell epitopes [29], which is a common strategy to reduce the immunogenicity of recombinant proteins for clinical use [30], and the humanized single-chain variable fragment against GPA33 (scFvA33) [31,32,33]. This target domain was already used in our previously developed immunotoxins [15,16,17,18,19,20]. The second was IMTXA33furαSDI, an optimized variant immunotoxin that also included a recognition and cleavage furin linker [34,35] between the marker and the toxic domain to improve the toxin release to the cytosol.

## 2. Materials and Methods

### 2.1. Plasmid Design

Plasmids containing the cDNA sequences that coded for IMTXA33αS and IMTXA33furαS were previously obtained. PCR was employed to amplify the sequences of interest using oligos designed to incorporate the desired mutations and the corresponding restriction sites. The resultant IMTXA33αSDI and IMTXA33furαSDI cDNA sequences were cloned into the plasmid pPICzαA (Invitrogen, Carlsbad, CA, USA) for the expression and extracellular translocation in the methylotrophic yeast *Pichia pastoris*. A 6xHis tag was included at the C-terminus of the proteins to facilitate their purification. The resulting plasmids (pPICZαAA33αSDI and pPICZαAA33furαSDI) were sequenced in order to confirm the desired sequence.

### 2.2. Cell Line Cultures

The SW1222 (ATCC HB-11028, Rockville, MD, USA) colon carcinoma cell line was used as a GPA33-positive cell line. The specificity of the target domain included in the immunotoxins described herein were previously characterized using other GPA33-positive or -negative cell lines [15,16,17,18,19,20]. Trypsinization was routinely carried out for harvesting and propagation of the SW1222 cultures. The THP1-xBlue cell line (Invivogen, San Diego, CA, USA) was used as a monocyte-like cell line. Upon activation via the NFkB pathway, the THP1-xBlue cells released an inducible form of secreted alkaline phosphatase reporter (SEAP). Peripheral blood mononuclear cells (PBMCs) from healthy donors were obtained and isolated from whole blood using Ficoll-Paque density gradient centrifugation. Both the THP1-xBlue cells and PBMCs were harvested and propagated by using centrifugation and dilution. All cells were grown in RPMI 1640 medium (Sigma-Aldrich, St. Louis, MI, USA), supplemented with glutamine (300 mg/mL), penicillin (50 U/mL), streptomycin (50 mg/mL), and zeocin (100 μg/mL) for the culture of the THP1-xBlue cells. Incubation of all cells was maintained at 37 °C in a humified atmosphere (CO_2_: air, 1:19, *v*:*v*). A Neubauer chamber was used to count the cells in all assays described.

### 2.3. Protein Production and Purification

Electrocompetent *P. pastoris* KM71H cells were electroporated with 10 μg of the linear plasmid (pPICZαAA33αSDI or pPICZαAA33furαSDI) using a Bio-Rad Gene pulser according to the device’s instructions. Multiple clones were isolated against media with different concentrations of zeocin (100, 400, or 750 μg/mL). To determine the best protein production colony and the optimal conditions for the immunotoxin production, a small production was first carried out. Different colonies were grown at 30 °C in 20 mL of BMGY for 24 h; then the cells were harvested and resuspended in 10 mL of BMMY for different times at 25 °C with vigorous shaking (200 rpm). The protein expression was then analyzed by using 0.1% (*w*/*v*) sodium dodecyl sulfate (SDS)–15% (*w*/*v*) polyacrylamide gel electrophoresis (PAGE) and Western blotting using a rabbit anti-α-sarcin antibody. The colony that produced the highest amount of IMTXA33αSDI or IMTXA33furαSDI was then selected for a large-scale production. For this purpose, 25 mL of preinoculum was added to baffled flasks containing 2 L of BMGY and incubated at 30 °C. After 24 h, the yeast was harvested and resuspended in 1 L of BMMY, and the protein production was induced at 25 °C with vigorous shaking. After 24 h, the extracellular medium was then dialyzed against 50 mM sodium phosphate buffer containing 0.1 M NaCl at pH 7.5. The purification of both immunotoxins was carried out via affinity chromatography using a Ni^2+^-NTA agarose column (GE Healthcare). The dialyzed medium containing the desired proteins was loaded into the column at a flow rate of 1 mL/min using a peristaltic pump. The column was then washed (first with sodium phosphate buffer and then with the same buffer with 20 mM imidazole added. The protein was then eluted with sodium phosphate buffer and imidazole (250 mM). Fractions were then analyzed via SDS-PAGE and Western blotting using a rabbit anti-α-sarcin antibody; the fractions in which the immunotoxin was detected were dialyzed against sodium phosphate buffer to remove the imidazole.

### 2.4. Structural Characterization

Structural characterization of the immunotoxins was performed via several means. Absorbance measurements were carried out on an UV-1800 spectrophotometer (Shimadzu, Shimadzu Europa GmbH, Duisburg, Germany). Far-UV circular dichroism (CD) spectra were obtained with a JASCO J-715 spectropolarimeter (Jasco Analítica, Madrid, Spain) at a scanning speed of 40 nm/min. Proteins were dissolved in PBS at a final concentration of 0.15 mg/mL, and 0.1 cm optical path cells were employed. At least six spectra were averaged to obtain the final data.

### 2.5. Ribonucleolytic Activity Assays

The specific ribonucleolytic activity of α-sarcin, which was included in the toxic domain of both IMTXA33αSDI and IMTXA33furαSDI, was analyzed as previously described [36,37] based on the detection of the characteristic 400 nt rRNA (known as the α-fragment) released from ribosomes from a rabbit cell-free reticulocyte lysate. First, the reticulocyte lysate was diluted threefold in Tris 40 mM and EDTA 10 mM pH 7.5 buffer, and aliquots of 50 μL were obtained and assayed against different amounts of the immunotoxin for 15 min at RT. The reaction was stopped by the addition of Tris 50 mM, SDS 5%, and pH 7.4 buffer. Then, RNA phenol/chloroform extraction was carried out, and the RNA was precipitated by the addition of isopropanol followed by resuspension in 10 μL of DEPC H_2_O. Release of the α-fragment was detected via electrophoresis on denaturing 2% agarose and 16% paraformaldehyde gels followed by ethidium bromide staining. Images were obtained with a Universal Hood II transilluminator.

### 2.6. Flow Cytometry Studies

Briefly, trypsinized SW1222 cells (GPA33+) were distributed into aliquots containing 3 × 10^5^ cells/mL and washed thoroughly three times with PBS containing 0.1% (*w*/*v*) of bovine serum albumin (BSA). The cells were then incubated with different concentrations of IMTXA33αSDI or IMTXA33furαSDI using gentle shaking for 30 min at RT. A second incubation was then performed by incubating the cells with the fluorescent-conjugated antibody anti-Histag-Alexa 488 (diluted 1:100; Sigma Aldrich, St. Louis, MI, USA) using gentle shaking for 30 min in the dark. Between the different steps, cells were collected via centrifugation (1200× *g* at 4 °C for 15 min) and washed with PBS and BSA 1% (*w*/*v*). Flow cytometry acquisition was carried out on a FACScan (Becton Dickinson, NJ, USA), and data were analyzed using the FlowJo v10 software (FlowJo, Oregon, OR, USA).

### 2.7. MTT Viability Assay

In order to analyze the specific cytotoxic effect of immunotoxins against the tumoral cells, MTT viability assays were performed. SW1222 (GPA33+) cells were seeded at a density of 5 × 10^3^ cells/well and incubated at 37 °C for 24 h. The medium was then removed, and IMTXA33αSDI or IMTXA33furαSDI was added to the cells at a final volume of 200 μL of medium. Samples were then incubated for 24, 48, or 72 h. After the incubation time, MTT was added at 0.5 mg/mL for 4 h at 37 °C, then the formazan crystals were dissolved via the addition of 100 μL of DMSO:methanol 1:1 (*v*/*v*), and then the viability was determined via the measurement of O.D. at 595 nm. Cells incubated with the medium and without the addition of immunotoxin were used as the 100% viability control. All samples were quadrupled, and the IC_50_ was calculated as the dose of immunotoxin that caused a 50% decrease in the cell viability.

### 2.8. Immunological Assays

#### 2.8.1. Monocytic Activation

THP1-xBlue cells were seeded at a density of 2 × 10^6^ cells/well with different concentrations of immunotoxin or LPS (10 ng) added as a positive activation control for 24 h. Monocytic activation was measured by adding Quanti-Blue (InvivoGen, San Diego, CA, USA) as a substrate of SEAP in the supernatants and then incubating for 6 h followed by measuring the optical density at 620 nm [38].

#### 2.8.2. PBMC Proliferation Assay

Freshly isolated PBMCs were seeded at a density of 5 × 10^4^ cells/well and incubated at 37 °C in 5% CO_2_ for 24 h. Then, different concentrations of immunotoxin or LPS (10 ng) as a positive activation control or immunotoxin-free medium as a negative control were added, and the incubation was carried out for 72 h. Then, the PBMCs were counted using a Neubauer chamber, and responses were defined as a stimulation index (S.I.), where S.I: = mean counts of the test wells/mean counts of the negative activation control wells. In addition, supernatants were collected for cytokine analysis.

#### 2.8.3. Cytokine Analysis

Using the supernatants of the stimulated PBMCs, the concentrations of IFNγ, IL-4, and IL-10 were determined via ELISA according to the manufacturer´s protocol (Sigma-Aldrich).

### 2.9. In vivo Antitumor Assays

All animal procedures were performed with the approval of the Complutense University Animal Experimentation Committee and the Community of Madrid as regarded in the Royal Decree 53/2013. Balb/c nude male mice that were 7 weeks old were obtained from Harlan Laboratories (Barcelona, Spain) to evaluate the in vivo effect of IMTXA33αSDI or IMTXA33furαSDI against colorectal cancer-induced xenografts. Animals were kept and maintained in the Animal Facilities of the Centro Investigaciones Biológicas–Consejo Superior Investigaciones Biológicas (CIB-CSIC) in Madrid.

For each immunotoxin, mice were allocated into three different experimental groups (*n* = 5): PBS (phosphate-buffered saline), IMTX25 (treatment with 25 μg of immunotoxin per injection), or IMTX50 (treatment with 50 μg of immunotoxin per injection). Before the experiments, animals were given an adaptation period with free access to food and water for 7 days. Then, each animal received a subcutaneous injection into the right flank of 2 × 10^6^ SW1222 cells resuspended in 100 μL of PBS and 100 μL of Matrigel (BD Biosciences, San Jose, CA, USA). When the tumor reached a volume of 50 mm^3^, mice were injected intravenously either with PBS or with different doses of immunotoxin. Six doses every 48 h of PBS or two different amounts of immunotoxins were given. The tumor volume was routinely measured for 30 days using an external caliper and was calculated using the following formula:

volume = width^2^ × length × 0.52

At the end of the experiment or when the tumor volume reached 2500 mm^3^, the animals were sacrificed. A survival analysis was carried out using Kaplan–Meier representation.

### 2.10. Statistical Analysis

ANOVA with a post hoc analysis via the Student–Newman–Keuls test was used within each test to compare the results obtained with the different constructions for each concentration in the different assays. All values were expressed as arithmetic means ± sem (standard error of the media). Differences between experimental groups were considered statistically significant at *p* < 0.05.

## 3. Results

### 3.1. Generation, Production, and Purification of Immunotoxins

**Figure 1:**
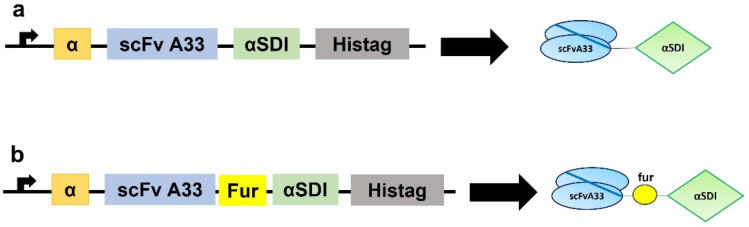
Schematic diagrams showing the genetic and protein domain arrangements of the immunotoxins IMTXA33αSDI (**a**) and IMTXA33furαSDI (**b**). The cDNA constructs appear on the left side of the figure, while the schematic representations of the protein domains are shown on the right side. In both cases, structural and functional domains are highlighted with different colors: α-factor secretion signal peptide (α; orange), scFvA33 (blue), furin linker (fur; yellow), non-immunogenic α-sarcin (αSDI; green), and histidine-tag (Histag; black).

Both expression vectors (pPICZαAA33αSDI and pPICZαAA33furαSDI) were obtained as described in Section 2 (Figure 1). After electroporation, both immunotoxins (IMTXA33αSDI and IMTXA33furαSDI) were successfully produced in the methylotrophic yeast *P. pastoris*.

**Figure 2:**
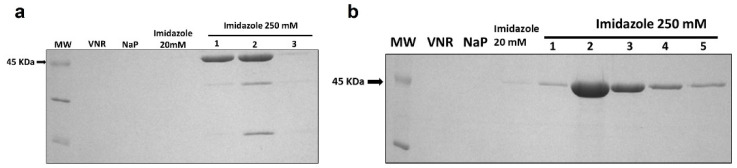
Coomassie-Blue-stained SDS-PAGE analysis of the different pools obtained during the Ni^2+^-NTA affinity chromatography of IMTXA33αSDI (**a**) and IMTXA33furαSDI (**b**). Lines shown correspond to: MW, prestained molecular weight standard (KDa); VNR, fraction not retained; NaP, washed fraction eluted with sodium phosphate buffer; Imidazole 20 mM, washed fraction eluted with sodium phosphate buffer containing imidazole 20 mM; and different 1 mL fractions eluted with 250 mM imidazole. For both immunotoxins, the arrow indicates the expected molecular weight of 45 KDa. The uncropped Western Blot images can be found in Figure S1.

**Figure 3:**
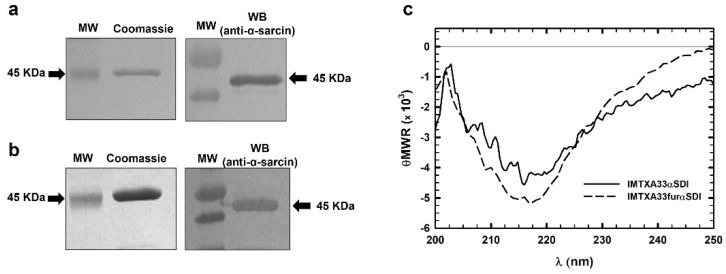
SDS-PAGE followed by Coomassie Blue staining and Western blot analysis of the purified final fractions of IMTXA33αSDI (**a**) and IMTXA33furαSDI (**b**). The Western blot analysis was carried out using rabbit anti-α-sarcin serum. MW corresponds to prestained molecular weight standards. Images were acquired and analyzed using the Quantity One 1-D analysis software (BioRad). In all cases, the arrows show the expected molecular weight of 45 KDa. (**c**) Structural characterization based on the far-UV CD spectra of IMTXA33αSDI (solid line) and IMTXA33furαSDI (dashed line). θMRW represents the mean residue weight ellipticities as degree × cm^2^ × dmol^−1^. Both spectra were obtained at a protein concentration of 0.15 mg/mL in 50 mM sodium phosphate and 0.1 M NaCl (pH 7.4). The uncropped Western Blot images can be found in Figure S2.

Purification was carried out from the extracellular medium followed by dialysis and immobilized metal affinity chromatography. Visualization of the purified immunotoxin was carried out via SDS-PAGE followed by Coomassie Brilliant Blue staining (Figure 2). Immunotoxins were present in the elution fractions at an expected molecular weight of 45 KDa; some cases showed lower molecular weight bands due to a slight degradation. The elution fractions were collected and the homogeneities and identities of IMTXA33αSDI and IMTXA33furαSDI were confirmed via SDS-PAGE followed by Coomassie Blue staining and immunodetection using an anti-α-sarcin antibody (Figure 3A,B). The final yields were 1 mg/L of induction (IMTXA33αSDI) and 0.9 mg/L (IMTXA33furαSDI).

### 3.2. Structural Characterization

The far-UV circular dichroism (CD) spectra of both IMTXA33αSDI and IMTXA33furαSDI were compatible with water-soluble proteins showing a high content of β-sheet secondary structure (Figure 3C), which was coherent for the predominant secondary structure of their structural components, the scFvA33, and the α-sarcin ribotoxin. 

### 3.3. In Vitro Functional Characterizations of IMTXA33αSDI and IMTXA33furαSDI

**Figure 4:**
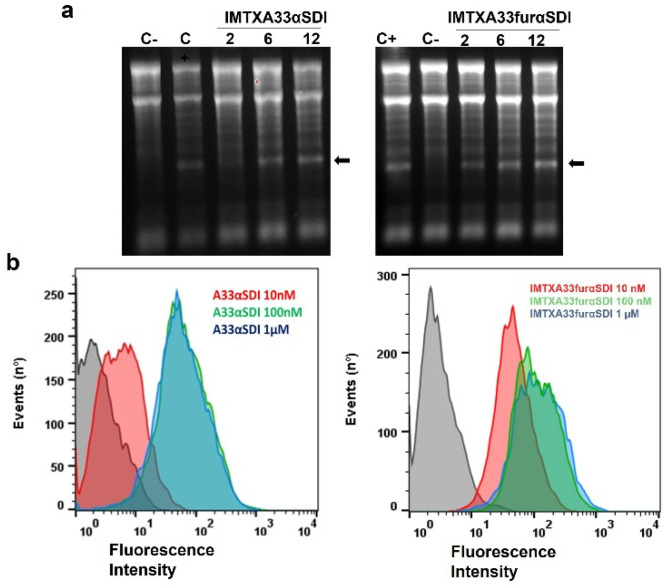
In vitro functional characterization. (**a**) Ribonucleolytic activity of the toxic domain of both immunotoxins as determined by a rabbit reticulocyte assay. The arrow indicates the release of the α-fragment produced by the cleavage of the SRL due to the αSDI. In both gels, 2, 6, and 12 pmoles of both IMTXA33αSDI and IMTXA33furαSDI were assayed. C+ represents 2 pmoles of fungal wild-type α-sarcin, whereas in C−, the protein sample was replaced with buffer. Images were acquired and analyzed using the Gel Doc XR Imaging System and the Quantity One software (BioRad). (**b**) Binding assays via flow cytometry of the targeting domain (scFvA33) of both IMTXA33αSDI and IMTXA33furαSDI with SW1222 (GPA33-positive cells). Curves correspond to cells incubated with the secondary antibody anti-His-Alexa488 (black) or 10 nM (red), 100 nM (green), or 1 μM (blue) of each immunotoxin. Fluorescence intensity was expressed in arbitrary units. The original full-legth gels can be found in Figure S3.

To analyze the in vitro activity of the new immunotoxins, the functionality of both the toxic domain and the binding domain was first assessed separately. The specific ribonucleolytic activity of the non-immunogenic α-sarcin, which was the toxic domain of both IMTXA33αSDI and IMTXA33furαSDI, was evaluated using the release and detection of the α-fragment from ribosomes in a reticulocyte cell lysate. Both IMTXA33αSDI and IMXA33furαSDI retained the ribonucleolytic activity of the ribotoxin by releasing the α-fragment and showed similar activity to WT α-sarcin (Figure 4A) in a dose-dependent ribonucleolytic activity.

The ability of the targeting domain (the scFvA33) for both immunotoxins was also assayed against the GPA33+ cell line (SW1222) via flow cytometry. As expected, both IMTXA33αSDI and IMTXA33furαSDI were able to bind specifically to the GPA33+ cell line in a similar way at a saturation binding concentration of 100 nM (Figure 4B).

**Figure 5:**
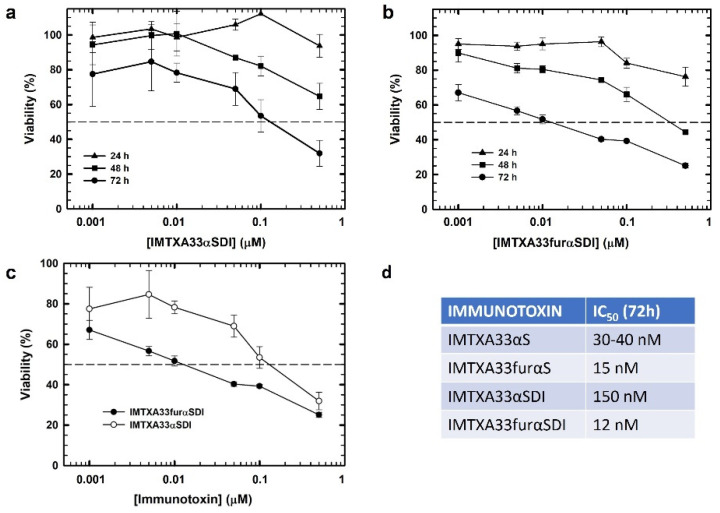
In vitro cytotoxic characterization. Viability assays via MTT were carried out against (GPA33-positive) SW1222 cells using IMTXA33αSDI (**a**) and IMTXA33furαSDI (**b**) at different times of 24, 48, or 72 h. (**c**) Differences in viability against SW1222 cells at 72 h for IMTXA33αSDI (open circles) or IMTXA33furαSDI (black circles). (**d**) IC_50_ values of different immunotoxins previously characterized at 72 h. Measurements were analyzed and plotted (mean ± SD) compared to untreated controls. In all cases, triplicate samples were carried out. IC_50_ values were obtained as the protein concentration that led to a 50% viability.

In vitro viability assays using MTT were performed to analyze the cytotoxic effect on tumoral cells due to the combinatorial effect of both the target domain and toxic domain of the immunotoxins. Both IMTXA33αSDI and IMTXA33furαSDI showed an efficient cytotoxic effect against the SW1222colorectal cancer cell line that depended on the immunotoxin concentration and time of incubation (Figure 5A,B). In addition, after 72 h of incubation, IMTXA33furαSDI exhibited an IC_50_ of 12 nM, which was 10 times lower than the IC_50_ displayed by IMTXA33αSDI (150 nm) (Figure 5C). This increase in the cytotoxic efficiency had already been described for the previously characterized IMTXA33furαS in comparison with IMTXA33αS (Figure 5D) [20].

### 3.4. Immunological Characterization of Immunotoxins

**Figure 6:**
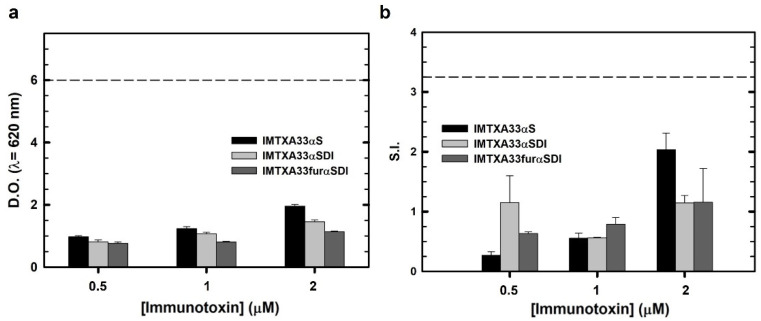
Immunological cell characterization. (**a**) THP1-xBlue cell activation assay. Different concentrations of IMTXA33αS (black), IMTXA33αSDI (white), and IMTXA33furαSDI (grey) were tested against THP1-xBlue cells. The Y-axis represents the optical density at 620 nm. The dashed horizontal line represents the activation threshold caused by LPS (10 ng). (**b**) Stimulation assay of PBMCs cultured with different concentration of IMTXA33αS (black), IMTXA33αSDI (white), and IMTXA33furαSDI (grey). Activation and proliferation of PBMCs was represented as the stimulation index (S.I.) (number of PBMCs for each treatment/number of PBMCs in the absence of immunogenic stimulus). The dashed horizontal line represents the activation threshold caused by LPS (10 ng).

Immunological characterization was performed via different assays to obtain an overall view of the immunogenic nature of the immunotoxins. In all experiments, both the non-immunogenic immunotoxins (IMTXA33αSDI and IMTXA33furαSDI) and the previously characterized immunotoxin (IMTXA33αS containing the wild-type α-sarcin as the toxic domain) [15] were assayed. First, THP1-xBlue reporter cells were incubated with the different immunotoxins in order to assess the response of this monocytic cell line. None of the immunotoxins assayed caused activation compared with the positive control LPS, and the “wild type” immunotoxin IMTXA33αS showed a slightly higher response compared with the non-immunogenic immunotoxin at the same concentration (Figure 6A).

These results were similar when the immunotoxins were cultured with PBMCs, so their proliferation was measured and made concrete as a stimulation index (S.I.), which is a suitable method to address the immunogenic nature of recombinant proteins [39]. None of the immunotoxins caused proliferation of the PBMCs above the threshold determined by LPS or above an S.I. value of 2.5, which is usually considered as a positive activation. Similarly to the monocytic activation essay, the wild-type immunotoxin (IMTXA33αS) caused the highest proliferation response, but this proliferation did not reach the activation threshold (Figure 6B).

**Figure 7:**
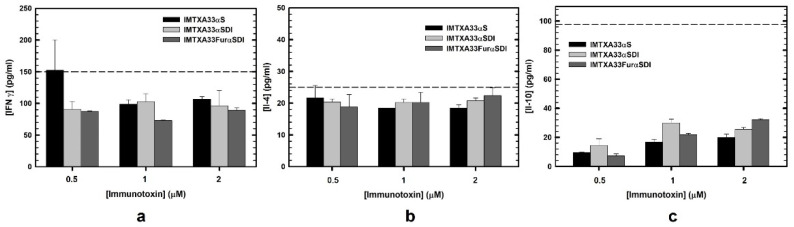
Cytokine quantification secreted by PBMCs and analyzed via ELISA in the presence of the different immunotoxins: IMTXA33αS (black), IMTXA33αSDI (white), and IMTXA33furαSDI (grey). IFNγ (**a**), IL-4 (**b**), and IL-10 (**c**) were the cytokines selected for the quantification via ELISA. The dashed line corresponds to the activation threshold representing the cytokine secretion caused by LPS (10 ng). Measurements were analyzed and plotted (mean ± SD) compared to untreated controls. In all cases, triplicate samples were carried out.

Furthermore, we analyzed the response in terms of cytokine production of PBMCs from healthy donors in the presence of the immunotoxins. So, supernatants of PBMCs cultured with the immunotoxins were collected, and quantification of IFNγ, IL-4, and IL-10 was carried out via ELISA. The selection of these cytokines was considered for their important role in Th1 and Th2 lymphocyte response or anti-inflammatory activity [40,41,42]. As can be observed in Figure 7, neither of the immunotoxins assayed produced a high cytokine secretion as compared to the positive control. 

### 3.5. In Vivo Antitumor Effect

**Figure 8:**
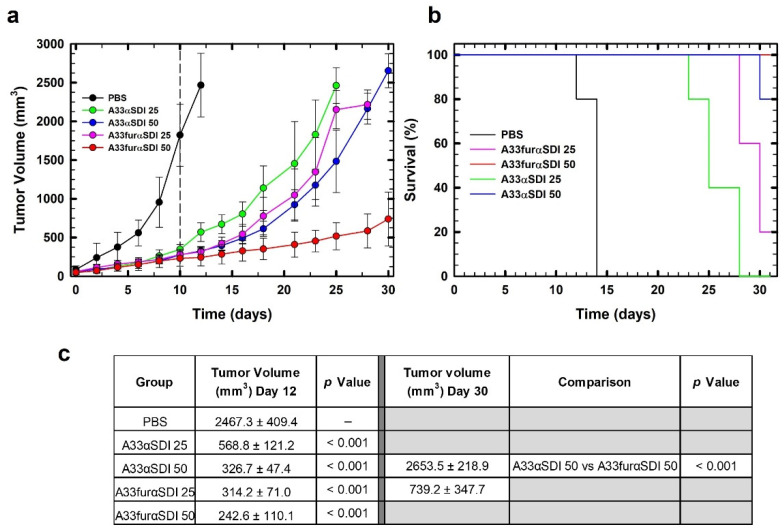
In vivo antitumoral activity. (**a**) Time course of tumor volume growth of SW1222-derived xenografts. Mice were non-treated with PBS (black) or treated with two different doses (25 or 50 µg) of IMTXA33αSDI (labeled in the graph as A33αSDI 25 (green) or A33αSDI 50 (blue)) or IMTXA33furαSDI (labeled in the graph as A33furαSDI 25 (pink) or A33furαSDI 50 (red)) per injection. The different doses were administered every 48 h. The vertical dashed line indicates the end of the treatment. (**b**) Kaplan–Meier survival curves. The Kaplan–Meier representation expressed the time to the experimental endpoint (once the tumor volume reached 2000 mm^3^ in the in vivo assay). The labels in the graph are the same as those used in (**a**). In all cases, the experimental groups were composed of 5 mice (*n* = 5). (**c**) Statistical analysis of IMTXA33αSDI- and IMTXA33furαSDI-treated tumors vs. vehicle-treated tumors at the end of the treatment (day 12) as well as IMTXA33αSDI-50- and IMTXA33furαSDI-50-treated groups (day 30).

Once we observed the in vitro cytotoxic activity of IMTXA33αSDI and IMTXA33furαSDI against SW1222 cells, the in vivo antitumor effect of these immunotoxins was analyzed using nude mice that bore subcutaneous SW1222 xenografts. When administered intravenously, both IMTXA33αSDI and IMTXA33furαSDI produced a strong inhibition of the tumor growth that resulted in a final tumor volume reduction of 7 times for 50 μg of IMTXA33αSDI and 10 times for 50 μg of IMTXA33furαSDI compared with the PBS group (day 12). After the administration of the immunotoxins, the tumor recovered its tumoral growth that was delayed when higher doses of immunotoxin were administered. Interestingly, the highest dose of IMTXA33furαSDI (50 μg) produced a significant reduction in the tumoral volume, and the growth was greatly reduced when compared with the other experimental groups (it reached only 700 mm^3^ by the end of the experiment (Figure 8A)).

These results were coherent when survival was analyzed using Kaplan–Meier representation. The survival rate was increased when higher doses of both IMTXA33αSDI and IMTXA33furαSDI were assayed; interestingly, the experimental group treated with 50 μg of IMTXA33furαSDI had an overall survival of 100% (Figure 8B).

## 4. Discussion

Immunotoxins are chimeric proteins that target TAAs that are differentially expressed on the surface of tumoral cells and are able to specifically kill them due to their toxic moiety [10,43]. In spite of their appealing designs, when immunotoxins have been tested in patients in clinical trials, several drawbacks have occurred that must be addressed. The first is the choice of TAA and the target domain in order to avoid off-target toxicities [7,11]. GPA33 is a well-known colorectal cancer marker that is overexpressed in 95% of colorectal cancer but absent from other healthy tissues. In addition, the scFvA33 that targets GPA33 is derived from a humanized mAb, and its binding properties and pharmacokinetics have been extensively described [15,16]. 

Another issue that requires consideration is the immunogenicity of the immunotoxins because the recognition of the therapeutic protein by the immune system of the patient can lead not only to a decrease in its therapeutic effect but also to inflammatory and autoimmune diseases. Among the different methods to reduce the immunogenicity of recombinant immunotoxins (such as PEGylation [44] or co-administration with immunosuppressive agents such as pentostatin or cyclophosphamide [45]), the elimination of T-cell epitopes has arisen as the best option because it reduces the immunogenic response of both B and T cells [30,46].

We produced, purified, and characterized a non-immunogenic variant of the previously characterized IMTXA33αS immunotoxin. As the target domain, the scFvA33 has already been humanized in order to prevent the antidrug antibody response of patients, the only moiety that remained immunogenic was the toxic domain (the α-sarcin). Even if fungal ribotoxins have already been described as less immunogenic than bacterial or plant toxins, the D9T and Q142T mutations have been reported to constitute a non-immunogenic variant of α-sarcin (αSDI) that lack the T-cell epitopes [29]. When αSDI is used as the toxic domain, the resulting immunotoxin (IMTXA33αSDI) is considered a whole non-immunogenic immunotoxin. 

In addition, the intracellular pathway of immunotoxins is considered of utmost importance due to its therapeutic effect [11,47]. In this sense, we produced, purified, and characterized an optimized variant of the non-immunogenic immunotoxin (IMTXA33furαSDI) that incorporated a furin recognition and cleavage linker between the target and the toxic domain that allowed the release of the toxic domain (αSDI), which led to a faster and more efficient tumoral death [20,48]. Because the secretion of immunotoxins in *P. pastoris* depends on the action of Kex2 proteases, which belong to the same protein family as furin, we selected a minimal recognition cleavage that consisted of Arg-X-X-Arg instead of the canonical sequence of Arg-X-(Arg/Lys)-Arg [34,49].

Both immunotoxins (IMTXA33αSDI and IMTXA33furαSDI) were produced in the methylotrophic yeast *P. pastoris* and purified via affinity chromatography at final yields of approximately 1 mg/L of induction. Both immunotoxins presented the expected size of 45 KDa and proper folding according to the structural characterization, which consisted of a majority β-sheet structure with slight differences between both immunotoxins, which possibly was due to the inclusion of the furin linker in IMTXA33furαSDI.

The functional characterization showed that both immunotoxins retained their ribonucleolytic activity and the binding ability of the marker domain to GPA33+ cells. This binding activity was also similar to that described for the original immunotoxins IMTXA33αS [15] and IMTXA33furαS [20], which indicated that scFvA33 is a suitable marker domain for the design of immunotoxins. Moreover, both were cytotoxic to SW1222 cells; IMTXA33furαSDI showed an IC_50_ of 12 nM, which was 10 times lower than the IC_50_ of IMTXA33αSDI, which was similar to the IC_50_ of the previously characterized “wild type” immunotoxin. The increased cytotoxic efficiency of IMTXA33furαSDI was mostly explained by the inclusion of the furin cleavage site. Once IMTXA33furαSDI was internalized in the early endosomes, the protease furin, which also was present in these endosomes, cleaved the immunotoxin and released the toxic domain. Once released, the αSDI could translocate to the cytosol and exert its antitumoral activity due to the ability of the toxin to interact with acid phospholipids located in the membranes of the endosomes [50,51]. In the absence of the furin cleavage site, after the internalization, the IMTXA33αSDI followed the endosome–Golgi apparatus network and was released later into the cytosol. As previously characterized, the inclusion of a furin linker increased the cytotoxic efficiency of immunoconjugates [20,35], which confirmed that the intracellular route followed by therapeutic proteins was one of the key optimization steps in the immunotoxin design.

Another crucial element of the therapeutic effect of immunotoxins is their immunogenicity. The elimination of immune cell epitopes is a prerequisite for their clinical application. Among immune cells, it has been well documented that the elimination of T-cell epitopes is a better strategy for reducing immunogenicity than the elimination of B-cell epitopes [52,53]. In this sense, both immunotoxins consisted of a T-cell-deimmunized variant of α-sarcin [29] as well as a humanized target domain. When these immunotoxins were assayed against the monocyte-like cell line or against human PBMCs, none of the immunotoxins (neither the non-immunogenic immunotoxins nor the original immunotoxin IMTXA33αS) triggered an activation of these immune cells. The low immunogenicity of IMTXA33αS could be inferred by the results described in [29], in which only 20% of the donor cohort elicited a positive response in a whole protein ex vivo DC:T cell assay after incubation with the α-sarcin wild type, which was negative in the case of αSDI (D9T/Q142T). Although the original construct did not reach the activation limit in the assays with the monocyte cell line and with PBMCs, a significant reduction in the response was observed with the non-immunogenic variants, especially at the highest concentration assayed (Figure 6).

To further analyze the interaction between the immunotoxins and PBMCs, the cytokine profile secretion of these PBMCs was determined. We selected IFNγ as a model cytokine produced by Th1 lymphocytes against bacteria and virus antigens [54]; IL-4 as a model cytokine secreted by Th2 lymphocytes against parasites and involved in allergic processes; and IL-10 as a pleiotropic cytokine but mostly involved in immunosuppressive functions [55]. In all cases, neither of the immunotoxins elicited any significant secretion of cytokines, and there was a lower production in the case of the non-immunogenic immunotoxins (IMTXA33αSDI and IMTXA33furαSDI). IMTXA33αS at the smallest dose analyzed (0.5 μM) produced an IFNγ response above the activation control, which probably was due to the experimental device because the response was lower at higher concentrations. In this sense, it would have been interesting to measure the IL-2 secretion because it is a more specific T-cell-activation model cytokine [23]. In summary, the results shown here confirmed that the new designed and characterized immunotoxins (IMTXA33αSDI and IMTXA33furαSDI) were non-immunogenic should not trigger any immune responses in patients even if several doses of treatment are administered, which is a common feature in immunotoxin therapeutics because both moieties of the immunotoxins have been either humanized (target domain) or deimmunized (toxic domain).

The potential immune safety of both IMTXA33αSDI and IMTXA33furαSDI as well as the functionality of both domains also were tested at 37 °C and for long periods, which encouraged the analysis of the antitumor activity in vivo. We previously characterized the antitumor activity of IMTXA33αS and showed a significant decrease in the volume of colorectal tumors xenografted in mice [19]. Because the immunotoxins were designed to be non-immunogenic in humans but not in mice, a nude mouse model was used in the in vivo assay in order to prevent an immune response being triggered against the immunotoxins by the mice.

IMTXA33αSDI exhibited a significant decrease in the tumor volume while the therapeutic doses were administered in a similar way to IMTXA33αS; when we stopped the administration of immunotoxin, the tumor volume was recovered as time passed. Interestingly, IMTXA33furαSDI exhibited a more efficient decrease in the tumor volume; and remarkably, the tumor exhibited a significantly slower recovery of the tumor growth when we stopped administrating therapeutic doses (final volume of 700 mm^3^ compared to the 2500 mm^3^ of the tumor treated with IMTXA33αSDI at the same doses). The more efficient antitumor effect of IMTXA33furαSDI as compared to that of IMTXA33αSDI can be explained by the presence of the furin linker and the intracellular processing, which led to a higher rate of tumoral death. It would still be interesting to analyze the longer-term antitumor effect of both immunotoxins by measuring the tumor volume for longer periods after drug administration or even by using a colorectal cancer model of mice instead of tumors xenografted in mice.

It has already been described in the literature that a furin linker increases the in vitro cytotoxic activity of immunotoxins. However, this was the first time that this effect was observed in vivo, which suggested that the inclusion of these linkers in the immunoconjugates could improve the therapeutic effect in patients. In addition, the safety provided by the non-immunogenic nature of the immunotoxin provides a new therapeutic tool that could potentially be used against colorectal cancer.

## 5. Conclusions

The work described herein represents a step forward in the design of potential therapeutic immunotoxins based on a non-immunogenic variant of α-sarcin [29] that showed an increased antitumor efficacy due to the inclusion of a furin linker that improved the toxin release to the cytosol. The combination of these optimization strategies represents an important boost to their therapeutic application.

## Data Availability

All experimental data generated or analyzed during this study are included in the article.

## Supplementary Materials

The following supporting information can be downloaded at: https://www.mdpi.com/article/10.3390/cancers15041114/s1, Figure S1: Original SDS-PAGE of both purified immunotoxins; Figure S2: SDS-PAGE and Western blot analysis of both purified immunotoxins; Figure S3: In vitro functional characterization.
