# Supplementary material for: A New Optimized Version of a Colorectal Cancer-Targeted Immunotoxin Based on a Non-Immunogenic Variant of the Ribotoxin α-Sarcin
Source: Cancers (Basel). 2023 Feb 9;15(4):1114. doi: 10.3390/cancers15041114 (PMC9954630; doi:10.3390/cancers15041114)
Supplement: Supplementary file 1 [file cancers-15-01114-s001.zip › cancers-2189454-supplementary.pdf]

Supplementary Materials: A New Optimized Version of a Colorectal Cancer-Targeted Immunotoxin Based on a Non-immunogenic Variant of the Ribotoxin *α*-Sarcin

Javier Narbona, Rubén G. Gordo, Jaime Tomé-Amat and Javier Lacadena

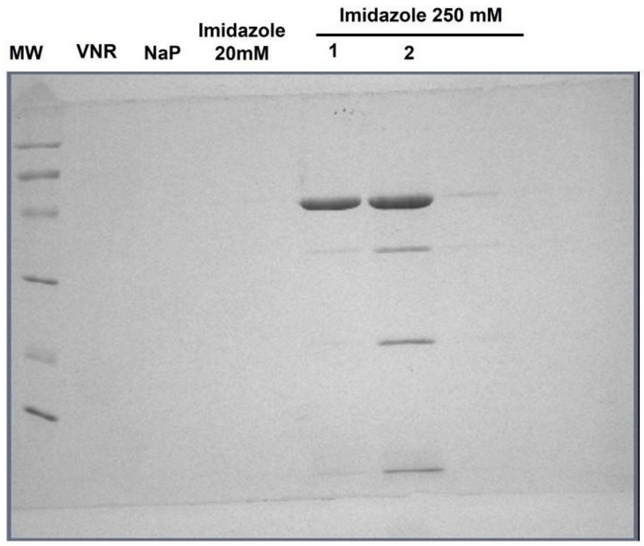

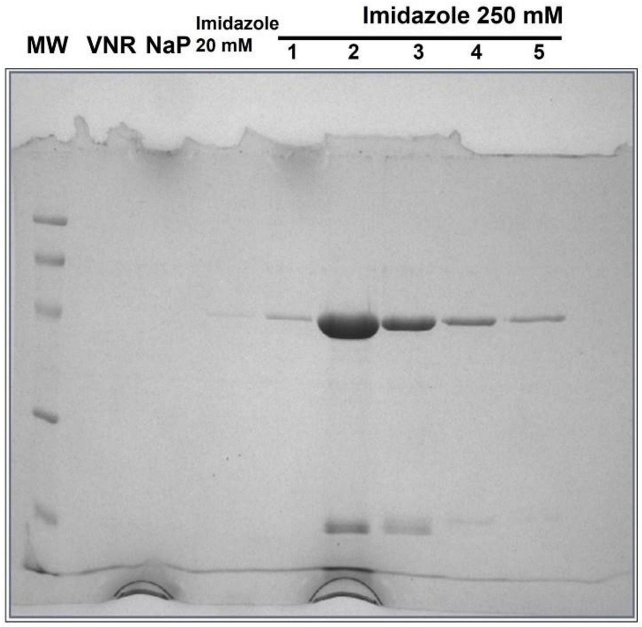

Figure S1. Original SDS-PAGE of both purified immunotoxins. Original full-length gels from Figure 2 are presented. Coomassie blue stained SDS-PAGE analysis of the different pools obtained during the Ni^{2+}-NTA affinity chromatography of IMTXA33*α*SDI (**a**) and IMTXA33fur*α*SDI (**b**). Lines shown correspond to: MW, prestained molecular weight standard (KDa); VNR, nor retained fraction; NaP, washed fraction eluted with sodium phosphate buffer; Imidazole 20 mM, washed fraction eluted with sodium phosphate buffer containing imidazole 20 mM; and different 1ml fractions eluted with 250 mM imidazole. In both immunotoxins, the arrow indicates the expected molecular weight of 45 KDa for both immunotoxins.

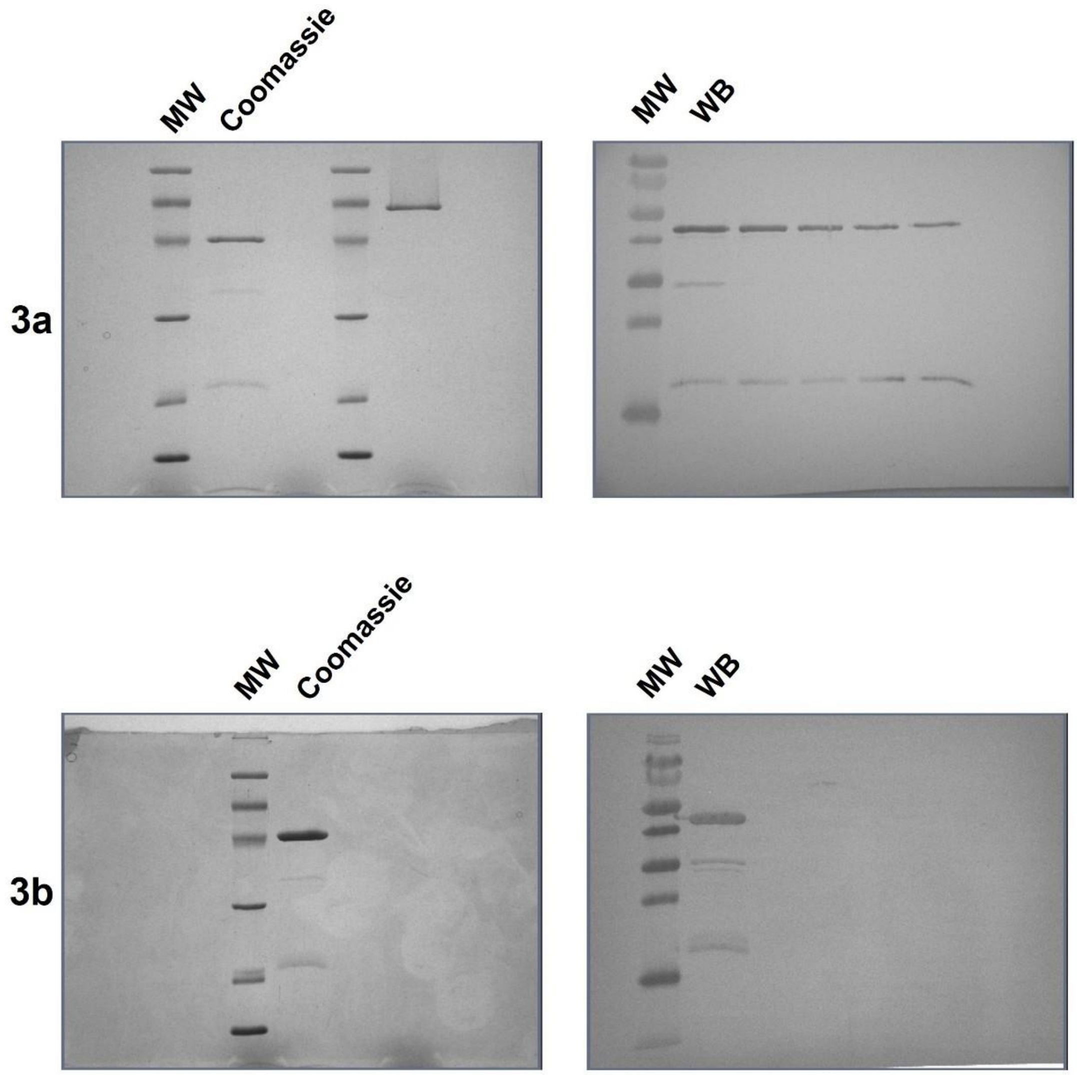

Figure S2. SDS-PAGE and Western Blot analysis of both purified immunotoxins. Original full-length gels and western blots from Figure 3a and 3b are presented. In all cases lines corresponding to information appeared in Figure 3 are indicated. SDS-PAGE followed by Coomassie blue staining (left) and Western Blot analysis (right) of the purified final fraction of IMTXA33αSDI. (**a**) and IMTXA33furαSDI (**b**). Western blot analysis was carried out using rabbitanti-α-sarcin serum. MW corresponds to prestained molecular weight standards. Images were acquired and analyzed using the Quantity One 1-D analysis software (BioRad).

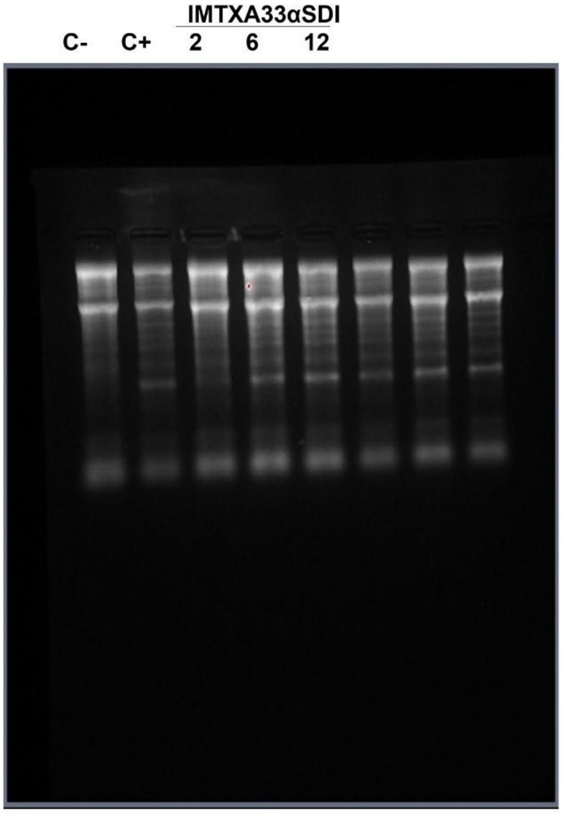

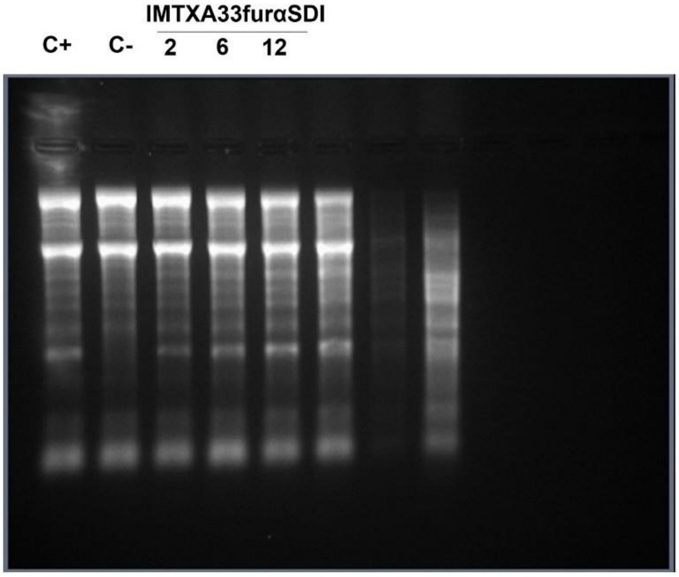

Figure S3. In vitro functional characterization. Ribonucleolytic activity of the toxic domain: Rabbit reticulocytes assays were made in order to test the ribonucleolytic activity of α-sarcin within both constructs. Original full-length gelsfrom Figure 4a are presented. (**a**) Ribonucleolytic activity of the toxic domain of both immunotoxins, carried out by a rabbit reticulocyte assay. The arrow indicates the release of the α-fragment, produced by the cleavage of the SRL due to the αSDI. In both gels, 2, 6 and 12 pmoles of both IMTXA33αSDI and IMTXA33furαSDI were assayed. C+ represents 2 pmoles of fungal wild-type α-sarcin, whereas in C–, the protein sample was replaced by buffer. Images were acquired and analyzed using the Gel Doc XR Imaging System and the Quantityone software (Bio-Rad).
